# Characterization of Uveal Melanoma Cell Lines and Primary Tumor Samples in 3D Culture

**DOI:** 10.1167/tvst.9.7.39

**Published:** 2020-06-29

**Authors:** Karen Aughton, Haleh Shahidipour, Luna Djirackor, Sarah E. Coupland, Helen Kalirai

**Affiliations:** 1 Liverpool Ocular Oncology Research Group, University of Liverpool, Liverpool, United Kingdom; 2 Translational Health Research Institute, Western Sydney University, Campbelltown, New South Wales, Australia; 3 Liverpool Clinical Laboratories, Liverpool University Hospitals Foundation Trust, Liverpool, United Kingdom

**Keywords:** uveal melanoma, spheroids, drug screening assay, drug penetration, personalized medicine

## Abstract

**Purpose:**

Uveal melanoma (UM) typically spreads to the liver, where it is incurable, as there are limited therapeutic interventions available. This study aimed to standardize laboratory methods for generating three-dimensional (3D) spheroids using UM cell lines and primary UM (PUM) samples for use in drug screening.

**Methods:**

Six UM cell lines and nine PUM, of differing genetic characteristics were cultured in two dimensions (2D) and three dimensions. 3D spheroid formation and growth were time monitored, and ImageJ software was used to calculate cross-sectional areas. PUM spheroids underwent immunohistochemistry for melanoma markers, nuclear BAP1, and cell proliferation. Chromosomal alterations in patient UM biopsies were compared with the corresponding 3D spheroid. In vitro drug assays testing doxorubicin and selumetinib assessed drug penetration and toxicity after 48 hours using imaging and the CellTiter-Glo 3D Cell Viability Assay.

**Results:**

All six UM cell lines formed spheroids of varying sizes and compactness; six of the nine PUM samples (67%) also formed spheroids, composed of MelanA+ proliferating melanocytes and admixed macrophages. PUM spheroids were genetically identical to the original sampled tumor. In vitro drug assays showed varying penetrations into UM cell line spheroids, with doxorubicin passing into the spheroid core and selumetinib having an effect largely on peripheral cells. Both drugs caused a dose-dependent reduction in viability of 3D spheroid cells.

**Conclusions:**

UM cell lines and PUM samples can successfully generate uniform 3D spheroids. PUM spheroids retain histological and genetic characteristics of the primary tumor. 3D spheroids are an important system for use in future high-throughput drug testing.

**Translational Relevance:**

The use of 3D spheroids allows early-phase drug screening and is an important first step toward treatment personalization for UM patients.

## Introduction

The increasing use of three-dimensional (3D) in vitro cell cultures in cancer is contributing to the development of more physiologically relevant models of tumor biology than standard two-dimensional (2D) cultures. 3D spheroids more closely recapitulate the physiology of the tumor microenvironment (TME), allowing cell-to-cell contact and cell-to-matrix synthesis,^1^ as well as the development of oxygen/nutrient gradients that occur across the spheroid.^2^ Cells remain in different proliferative and metabolic states within the spheroid, as would be expected within the tumor. 2D cultures also show artificially high levels of cell proliferation that are not typically seen in patient tissues, often resulting in enhanced drug sensitivities that are not representative of drug efficacy in vivo.^3^ In contrast, 3D spheroids have been shown to convey reduced drug sensitivities when compared with traditional 2D systems, suggesting that they could better model in vivo drug potencies, and this may help with testing new anticancer strategies.^4^

To date, in vitro studies examining uveal melanoma (UM) biology, such as cancer cell invasion and migration, TME signaling and crosstalk, and responses to anticancer therapies, have generally been conducted using 2D cultures of UM cell lines.^5^^–^^7^ The lack of effective tumor treatments in metastatic UM creates an urgent unmet need for improved cellular systems, not only to enhance our understanding of this disease but also to more accurately translate drug efficacy to patients. Recent studies have profiled more complex UM culture systems, including 3D spheroids created from UM cell lines embedded in either collagen or Matrigel (Corning Inc., Corning, NY)^8^^,^^9^; however, the methods used by these groups and the technical challenges, such as spheroid uniformity, are not described in great detail. Method optimization for 3D spheroid culture and an improved understanding of the advantages and challenges that each of these methods offers are key to bridging the gap between 2D-cultured monolayers, animal models, and patient clinical trials.

In this study, we provide detailed methods for cultivating 3D spheroids using a range of UM cell lines and, *for the first time*, to the best of our knowledge, human primary UM (PUM) samples. We describe the UM spheroid growth, their phenotype, use in drug screening assays, and the effect of two therapeutic agents (doxorubicin and selumetinib) used previously for metastatic UM on these spheroids.

## Materials and Methods

### 2D Cell Culture

#### Cell Lines

The following UM cell lines (stock passage number) were used in the studies: 92.1 (P57), OMM2.5 (P20), MM66 (P44), MP41 (P177), OMM1 (P+3), and MP46 (P26).^10^^–^^13^ All UM cell lines were authenticated by short tandem repeat (STR) profiling according to published data^14^ or data available from American Type Culture Collection. Cell lines were used within 10 passages of resuscitation and were free from mycoplasma. Cells were maintained under standard conditions (37°C, 5% CO_2_) in RPMI with GlutaMAX (Gibco, Thermo Fisher Scientific, Waltham, MA) supplemented with 10% fetal calf serum (FCS; Labtech, East Sussex, UK), hereafter referred to as UM medium A. For all studies, UM cell lines were harvested at ∼70% confluence with 0.05% trypsin/ethylenediaminetetraacetic acid (EDTA), counted and resuspended at varying cell densities (5000, 7500, 10000 cells/well) for 3D culture, as indicated below.

#### Primary UM

This study was approved by the Health Research Authority (HRA reference: 15/SS/0097), and all UM patients provided informed written consent for the use of their material and data in research. Fresh PUM specimens from patients undergoing enucleation or local resection were obtained from the Liverpool Ocular Oncology Biobank (LOOB) (HRA reference: 16/NW/0380). A small piece of fresh PUM tissue, approximately 3mm^3^, was finely minced with a sterile scalpel blade and digested with 500-U/ml type I collagenase (Sigma-Aldrich, St. Louis, MO) at 37°C for approximately 1 hour with occasional agitation, as previously described.^15^ Single cells were harvested by centrifugation at 1800 rpm for 2 minutes and resuspended in primary cell culture medium, hereafter referred to as UM medium B: α-MEM (Sigma-Aldrich) supplemented with AmnioSelect (Metachem Diagnostics, Northampton, UK), 10% FCS (Labtech), 2-mM l-glutamine (Sigma-Aldrich), and Gibco 1% penicillin–streptomycin (5000 U/ml, Thermo Fisher Scientific). Cells were maintained in a humidified incubator (5% CO_2_) at 37°C and harvested at 60% to 70% confluence with 0.05% trypsin–EDTA solution (Thermo Fisher Scientific); they were then counted and resuspended for 3D culture at varying cell densities (2000, 4000, 8000, or 16,000 cells/well), as indicated below.

### Spheroid Assay

UM cell lines and PUM cells were harvested as described above and plated into ultra-low attachment (ULA), 96-well, clear, round-bottomed plates (Corning) at varying densities in 100-µl volumes of UM medium A or medium B for cell lines and PUM cells, respectively. Cells were imaged between days 1 and 10 of incubation with the EVOS M5000 Imaging System (Thermo Fisher Scientific) or a Zeiss Axio Observer Z1 microscope (Carl Zeiss Meditec, Oberkochen, Germany).

#### Measurement of Spheroid Size

Spheroid images were analyzed for growth determination using ImageJ/Fiji software (National Institutes of Health, Bethesda, MD) and an open-source macro developed by Ivanov et al.^16^ The macro automated the measurement of the maximum spheroid cross-sectional area by converting images to black and white before applying the Yen thresholding algorithm.^17^ Data were subsequently collated and further analyzed using Excel (Microsoft Corp., Redmond, WA) and GraphPad Prism (GraphPad, San Diego, CA). For both PUM and cell lines, three to six spheroids were analyzed for each time point and, in the case of the UM cell lines, in three separate experiments.

### Immunohistochemistry

PUM spheroids were removed from the ULA plates using a cut 200-µl pipette tip at various time points and fixed in 10% neutral buffered formalin for 15 minutes. Spheroids were then suspended in 2% agar before being processed using the Bayer Tissue-Tek VIP E300 tissue processor (Bayer AG, Leverkusen, Germany). Processed spheroids were subsequently embedded in paraffin blocks and sectioned at 4 µm onto X-tra adhesive slides (Leica Biosystems, Wetzlar, Germany), for immunohistochemical (IHC) staining.

IHC staining was performed as previously described^18^ using the Dako Pre-Treatment Module and the Dako Envision FLEX kit (Agilent Technologies, Santa Clara, CA), according to the manufacturer's instructions. Details of antibodies, antigen retrieval, and concentrations are provided in Table 1. Positive staining was visualized with an AEC substrate kit (Vector Laboratories, Burlingame, CA) according to the manufacturer's instructions. Sections were counterstained with Mayer's hematoxylin (VWR, Leighton Buzzard, UK), dyed blue with Scott's tap water (Leica), and mounted using Aquatex aqueous mounting medium (Sigma-Aldrich). Slides were scanned using the Leica Aperio CS2 slide scanner at 20× magnification.

**Table 1.** Antibodies Used to Stain and Characterize Formalin-Fixed PUM Spheroids

Antibody	Species	Isotype	Dilution	Supplier	Positive Human Control Tissue
MelanA	Mouse	IgG1, mouse monoclonal	1:100	Agilent Technologies (Santa Clara, CA)	Eye containing UM
Ki67	Mouse	IgG1, mouse monoclonal	1:200	Leica Biosystems (Wetzlar, Germany)	Tonsil
BAP1	Mouse	IgG1, mouse monoclonal	1:200	Santa Cruz Biotechnology (Dallas, TX)	Pancreas
CD68	Mouse	IgG1, mouse monoclonal	1:200	Dako A/S (Glostrup, Denmark)	Tonsil

IgG1, immunoglobulin G1.

### Genetic Characteristics of UM Spheroids by MLPA

Clinical, histopathological, and genetic information for the PUM samples used in 3D culture was provided by the LOOB and is shown in Table 2. PUM spheroids were also assessed for copy number variations (CNVs) in chromosomes 1, 3, 6, and 8q by multiplex ligation-dependent probe amplification (MLPA). In brief, three PUM spheroids from each patient were pooled and lysed at day 9, and DNA was extracted using the DNeasy kit (QIAGEN, Hilden, Germany) according to previously published protocols.^19^ As previously described,^20^ 100 ng DNA was used for MLPA, and chromosomal alterations were compared with the patient tumor as displayed in Table 2.

**Table 2.** Patient Demographics of PUM Samples Used for 2D Cell Culture

	PUM								
Sample ID	S084	S093	S104	S119	S121	S143	S145	S006	S082
Clinical information								
Tumor location	Cilio choroidal	Cilio choroidal	Choroidal	Choroidal	Choroidal	Choroidal	Choroidal	Choroidal	Choroidal
Previous treatment	No	No	No	No	No	No	No	No	No
Histopathological information								
Specimen type	Enuc	Enuc	Endo	Enuc	Enuc	LR	Enuc	Enuc	Enuc
Cell type	Epithelioid	Spindle	Mixed	Mixed	Mixed	Epithelioid	Epithelioid	Spindle	Epithelioid
Presence of vascular loops	Yes	No	NA	Yes	Yes	NA	Yes	Yes	Yes
Mitotic count/40HPF	4	4	NA	3	2	2	14	3	38
Macrophage infiltration (density)	Mild	Mild	NA	Mild	Mild	Dense	Moderate	Mild	Moderate
nBAP1 protein expression	Negative	Positive	NA	Positive	Negative	Positive	Positive	Positive	Negative
Genetic information								
Chr3	L	N	N	N	L	N	L	N	L
Chr8q	N	G	N	N	N	N	G	G	G

Endo, endoresection; Enuc, enucleation; LR, local resection; 40HPF, 40× high-powered field; nBAP1, nuclear BAP1; L, loss; N, normal; G, gain; NA, not assessed.

### Drug Cytotoxicity Assays

UM cell lines 92.1 and MM66 were seeded at 5000, 7500, and 10,000 cells/well in ULA plates and grown for 4 days to form compact spheroids. The 92.1 and MM66 UM cells were also plated into 2D, flat-bottomed, 96-well plates at a density of 10,000 and 15,000 cells/well, respectively, for 24 hours to reach 70% confluence. For drug testing in both 2D and 3D, either doxorubicin or the MEK inhibitor selumetinib was added at doses between 0.5 and 10 µg/ml and between 0.03 and 30 µM, respectively, 24 hours after plating the cells in 2D, as well as on day 4 of spheroid formation, following imaging of each spheroid. The spheroids were imaged and both 2D and 3D cultures were assessed for cell viability 48 hours after drug addition, as described below.

### Cell Viability Assay

Cell viability was assessed in both 2D and 3D cultures using the CellTiter-Glo 3D Cell Viability Assay (Promega) according to the manufacturer's instructions using black opaque-walled multi-well plates suitable for luminescence measurements. In brief, 50 µl medium was removed from each well, and 50 µl CellTiter-Glo 3D was added. All procedures were performed at room temperature. The mixture was pipetted up and down for 30 seconds and assessed microscopically for complete lysis of the spheroid/cells before transferring to a black opaque-walled multi-well plate and incubating on a shaker in the dark for 5 minutes. After a further 25-minute incubation in the dark, luminescence was recorded using a Spark plate reader (Tecan Trading AG, Männedorf, Switzerland).

## Results

### Generation of PUM and UM Cell Line Spheroids

Use of the ULA plate methodology resulted in both PUM and UM cell lines successfully forming spheroids. Spheroids that formed from different PUM and UM cell lines were of varying compactness, size, and density, as described below.

#### Cell Lines

**Figure 1.**
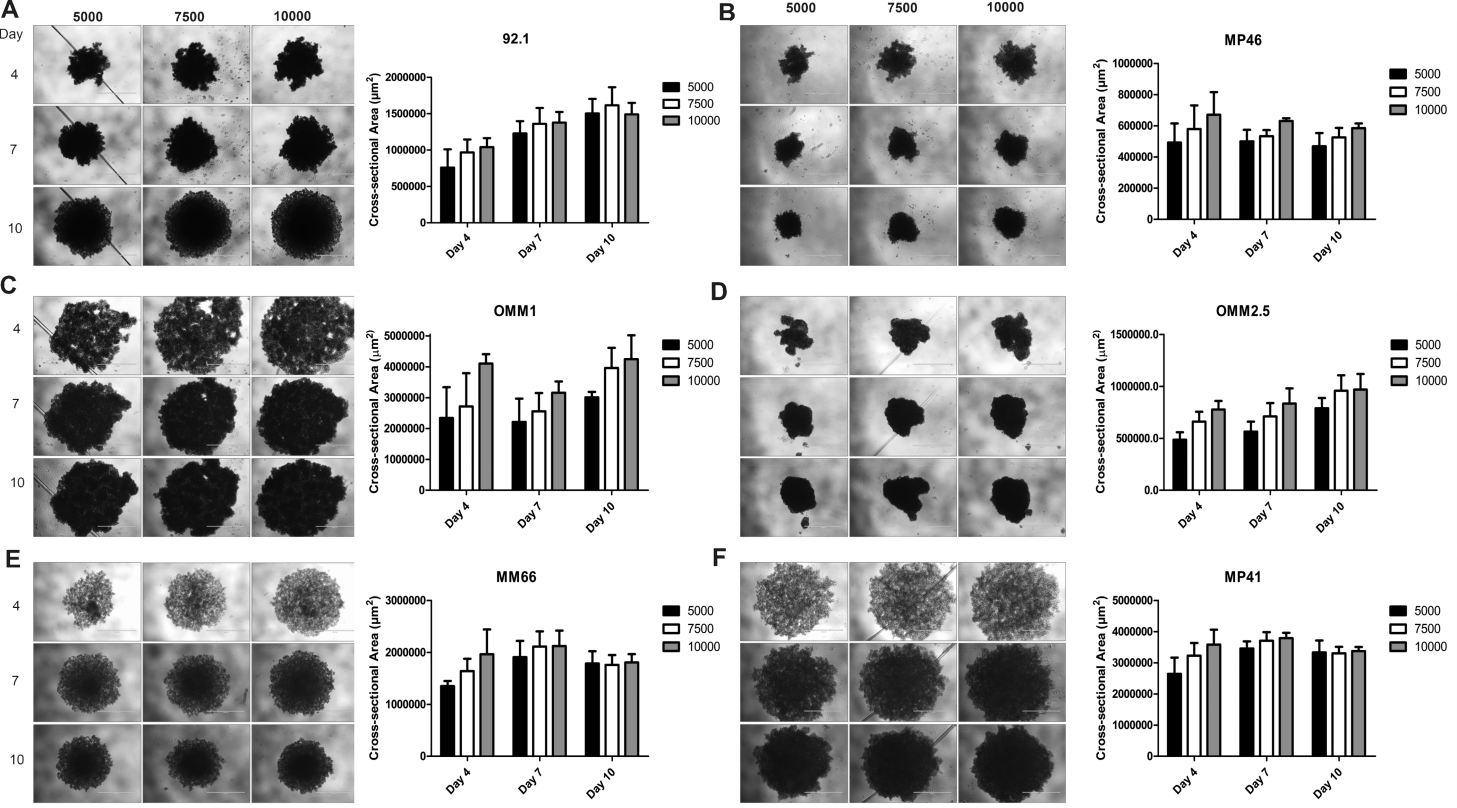
Representative 3D spheroids of UM cell lines seeded at 5000, 7500, and 10,000 cells per well at days 4, 7 and 10 with corresponding spheroid cross-sectional area measurements (4× magnification; n ≥ 6). UM cell lines (A) 92.1, (B) MP46, (C) OMM1, (D) OMM2.5, (E) MM66, and (F) MP41. Data are mean ± SD.

All UM cell lines tested successfully formed spheroids (Fig. 1). Cell line spheroids varied in compactness, with 92.1, OMM2.5, and MP46 cells forming small, tightly packed aggregates that could be easily manipulated by day 4. In contrast, MP41 and OMM1 cells formed loose aggregates, even at day 10, which were difficult to technically manipulate further, possibly due to limited cell-to-cell interaction. MM66 cells formed small spheroids of intermediate compactness. Each cell line differed in spheroid size between days 4 and 10, as measured by cross-sectional area, reflective of proliferation and compactness (Fig. 1). MP41 and OMM1 showed the greatest cross-sectional area (i.e., formed the largest spheroids) at all plating densities and time points, whereas MP46 and OMM2.5 produced small, dense spheroids. 3D spheroids formed by 92.1 and MM66 cells were intermediate in terms of cross-sectional area at the time points and plating densities examined; 92.1 had ruffle-like edges, which have been suggested by others to be associated with proliferating cells at the spheroid edge.^21^ The 92.1, OMM1, and OMM2.5 cells demonstrated a lag phase in spheroid growth, possibly due to continued compacting of cells, with a significant increase in spheroid cross-sectional area occurring for 92.1 and 0MM2.5 spheroids only by day 10 when compared with day 4 at all plating densities (*P* < 0.01, *t*-test). MM66 and MP41 spheroids demonstrated an initial growth phase up to day 7, which was followed by a reduction or stabilization of spheroid size by day 10, reflective of compaction occurring at this later time point. Statistically significant growth between day 4 and day 10 was only observed when MM66 and MP41 cells were plated at 5000 cells/well. MP46 cells showed no significant alteration in spheroid size between days 4 and 10 at any of the plating densities (*P* > 0.01, *t*-test), possibly due to the long doubling time of these cells (∼110 hours).

These spheroid characteristics were not associated with cell line tissue of origin (i.e., liver metastasis, subcutaneous metastasis, or primary ocular tumor) nor were there associations with cell morphology (i.e., spindle or epithelioid in shape) or the genetic profile of UM cells.

#### Primary UM

**Figure 2.**
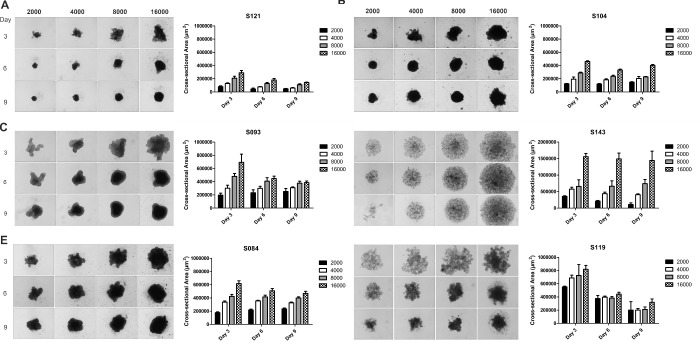
Representative 3D spheroids of PUM cells seeded at 2000, 4000, 8000, and 16,000 cells per well at days 3, 6 and 9 with corresponding spheroid cross-sectional area measurements (4× magnification; n = 3). PUM cells (A) S121, (B) S104, (C) S093, (D) S143, (E) S084, and (F) S119. Data are mean ± SD.

All PUM samples were successfully cultured in 2D for a single passage; however, three out of nine samples (S145, S006, and S082) then failed to grow and form 3D spheroids when added to ULA plates at varying cell densities. The histological and genetic characteristics of the nine samples are shown in Table 2, and there were no obvious morphological or genetic differences in the characteristics of the samples to explain why some formed spheroids and others did not. As seen with the UM cell lines, the PUM spheroids that formed from cells isolated from different tumors (S121, S093, S084, S104, S143, and S119) varied in shape, size, and compactness, despite being plated at similar cell densities (Fig. 2). In general, PUM at early time points and low seeding densities (2000 or 4000 cells) formed loose aggregates. Spheroid cross-sectional area was greatest at a density of 16,000 cells/well compared with 2000 cells/well on all days, as would be expected, but in general PUM spheroids, even at the highest plating density, were smaller than those formed by UM cell lines at similar time points and lower plating densities. Five of the six PUM samples (S121, S093, S084, S104, and S119) formed compact spheroids by day 9 (similar to 92.1, OMM2.5, and MP46 cells); however, the morphological features of S119 suggest that this was concomitant with some cell death. These spheroids could be further manipulated for fixation and IHC staining while retaining their structure. A single sample (S143) continued to grow as loosely packed cells similar to MM66 and MP41 UM cell lines. This was visible morphologically at all plating densities; however, S143 could not be handled for fixation and downstream applications.

**Figure 3.**
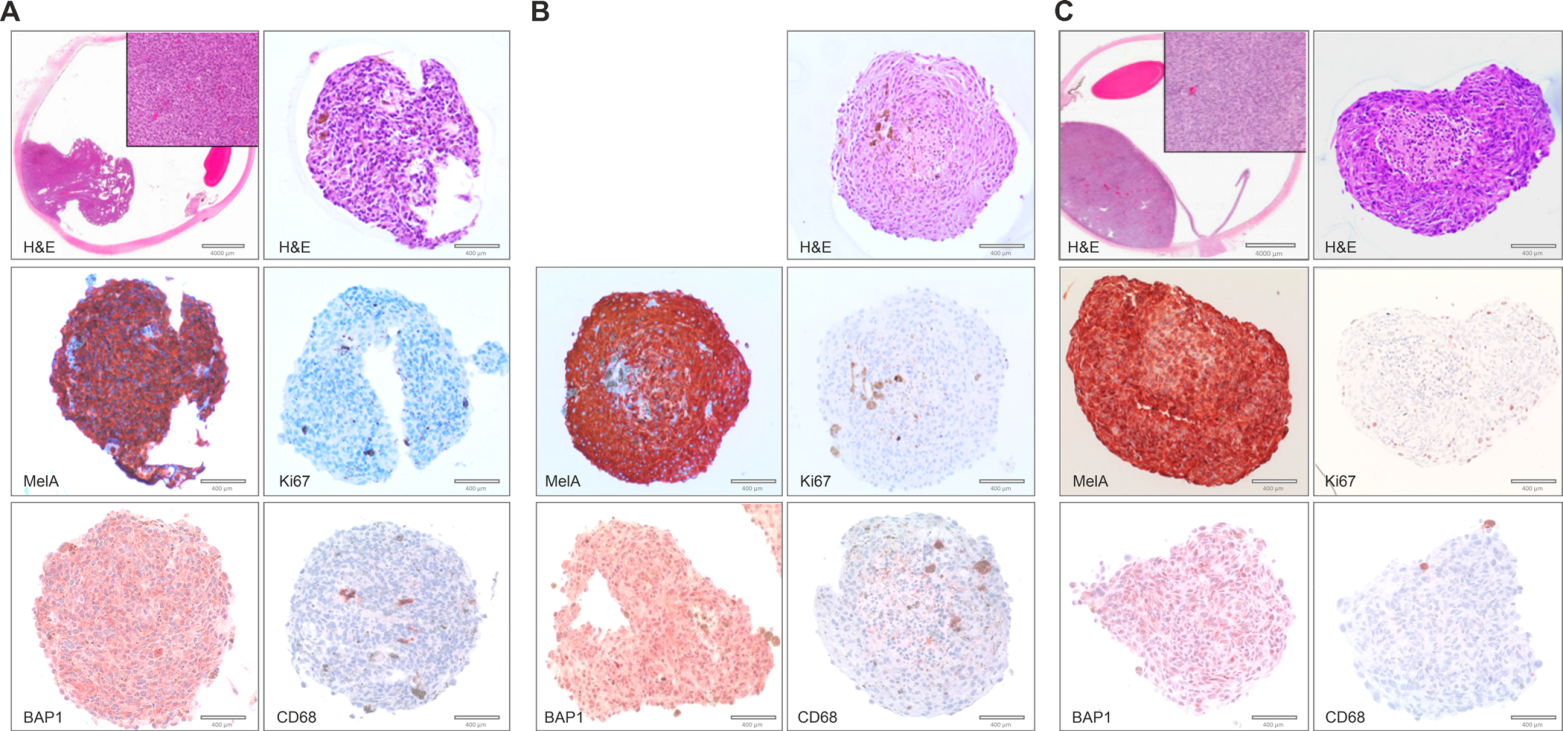
Immunohistochemistry of 3D primary UM spheroids stained with various antibodies (20× magnification). (A) PUM enucleation S121 stained for H&E, MelA, Ki67, BAP1, and CD68; (B) PUM endoresection S104 stained for H&E, MelA, Ki67, BAP1, and CD68; (C) PUM enucleation S093 stained for H&E, MelA, Ki67, BAP1, and CD68.

To determine whether there was genetic drift between the spheroids and the original sampled tumor tissue, MLPA was undertaken from both the original patient tissue and pooled PUM spheroids from S121, S093, S084, S104, and S119 at day 9. MLPA showed that the PUM spheroids retained the same CNVs compared to the PUM, with the exception of S121 in which chr6p and chr8q status differed from its counterpart PUM (Table 3). UM cell phenotype, nBAP1 status, and presence/absence of macrophages were all concordant between the PUM and the corresponding 3D spheroid at day 9 for cells plated at 8000 cells per well (Fig. 3, Table 3) and for all other plating densities analyzed (data not shown). Figure 3 shows representative IHC images from three PUM samples (S121, S104 and S093) of increasing cross-sectional area at day 9 when plated at 8000 cells per well, with mean ± SD of 108411.5 ± 15012.3 µm^2^, 227550 ± 7050 µm^2^, and 376890.5 ± 29881.3 µm^2^, respectively. Of note is the clear necrotic core observed in S093. This may be due to the proliferative nature of the cells in this sample as evidenced by numerous Ki67-positive cells at the periphery, which are largely absent in S121 and S104.

**Table 3.** Comparison of Histological and Genetic Characteristics of PUM Tissue and 3D Spheroid

PUM		Cell Type	Macrophages	nBAP1	Chr1	Chr3	Chr6p	Chr6q	Chr8p	Chr8q
S084	Patient tumor	Epithelioid	Mild	Negative	L	L	N	N	N	N
	3D spheroid	Epithelioid	Present	Negative	NA	NA	NA	NA	NA	NA
S093	Patient tumor	Spindle	Mild	Positive	N	N	G	L	N	G
	3D spheroid	Spindle	Present	Positive	N	N	G	L	N	G
S104	Patient tumor	Mixed	NA	NA	N	N	N	N	N	N
	3D spheroid	Spindle	Present	Positive	N	N	N	N	N	N
S119	Patient tumor	Mixed	Mild	Positive	N	N	G	N	N	N
	3D spheroid	Spindle	Present	Positive	N	N	G	N	N	N
S121	Patient tumor	Mixed	Mild	Negative	L	L	G	L	N	N
	3D spheroid	Spindle	Present	Negative	N	L	N	L	N	G

nBAP1, nuclear BAP1; L, loss; N, normal; G, gain; NA, not assessed.

### Drug Cytotoxicity Assays

Studies have previously shown that differing drugs will induce changes in spheroid morphology characteristic of the drug and its mechanism of action.^22^^–^^24^ We selected doxorubicin (topoisomerase 2 inhibitor), a commonly used cancer chemotherapeutic with autofluorescence properties that can be used to observe drug penetration throughout spheroids, and selumetinib as a targeted small molecule MEK inhibitor, previously used in clinical trials to treat metastatic UM with mixed results.^25^^,^^26^ Both 92.1 and MM66 UM cell lines were chosen to represent a primary and metastatic tumor phenotype, respectively, and they showed compacted cores at day 4 with visibly proliferating cells at the periphery of the spheroid. In addition, the differing growth characteristics of these two cell lines were representative of that observed in the PUM samples.

**Figure 4.**
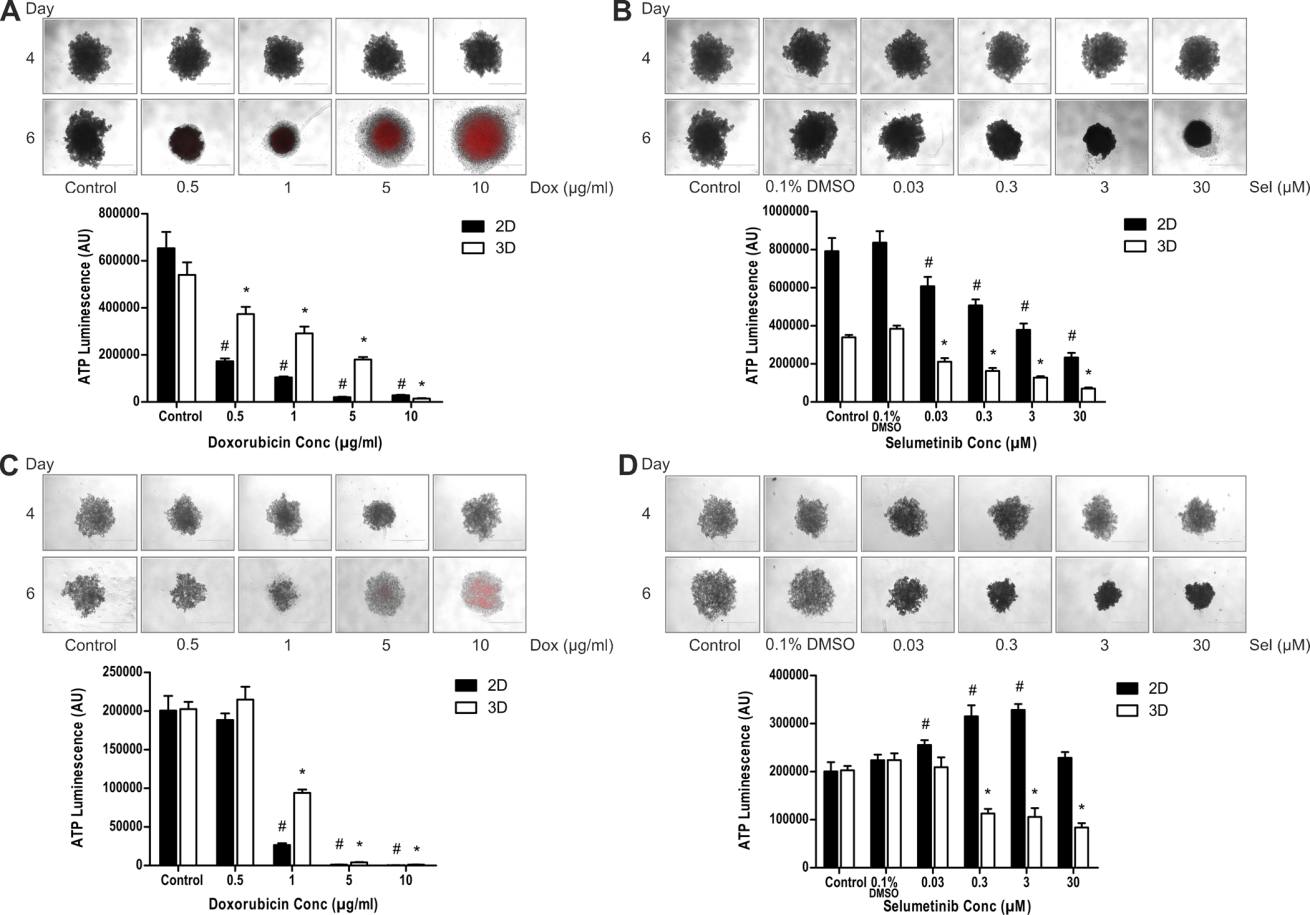
Drug cytotoxicity assays of 92.1 and MM66 UM cell line 3D spheroids treated with doxorubicin and selumetinib. Representative images of the spheroids from pre-dosing (day 4) and 48 hours after dosing (day 6) are shown (4× magnification; n = 6). Cell viability was assessed using the CellTiter-Glo 3D Cell Viability Assay. (A) 92.1 and doxorubicin; (B) 92.1 and selumetinib; (C) MM66 and doxorubicin; (D) MM66 and selumetinib. Data are mean ± SD. Values significantly different (*P* < 0.01, *t*-test) from control (doxorubicin) or 0.1% DMSO (selumetinib) are denoted with # for 2D and * for 3D.

Following a 48-hour exposure, doxorubicin was shown to penetrate through both 92.1 and MM66 spheroids at higher concentrations (≥5 µg/ml). This resulted in dispersion and disaggregation of the cells with increased cross-sectional area of the spheroids in both cell lines (Figs. 4A, 4C). Increasing doxorubicin concentrations were accompanied by significantly (*P* < 0.01, *t*-test) decreased adenosine triphosphate (ATP) levels and hence spheroid cell viability. For 92.1, this was first seen at 0.5 µg/ml with a 31% reduction in viability; for MM66, at 1.0µg/ml with a 54% reduction in viability when compared with the untreated control (Figs. 4A, 4C). The highest concentration of doxorubicin tested (10 µg/ml) reduced spheroid cell viability by ≥99% in both cell lines when compared with untreated control. Similar dose–response curves were observed for 92.1 and MM66 cells treated with doxorubicin in 2D culture (Figs. 4A, 4C).

Selumetinib caused a decrease in spheroid cross-sectional area at drug concentrations ≥ 0.03 µM for 92.1 spheroids, and ≥ 0.3 µM for MM66 spheroids (Figs. 4B, 4D). This was further supported by the significant (*P* < 0.01, *t*-test) decrease in ATP measurements and hence spheroid cell viability at these drug concentrations when compared with the 0.1% dimethyl sulfoxide (DMSO) control. For 92.1, there was a 45% reduction in spheroid cell viability and for MM66 a 50% reduction in spheroid cell viability when compared with the 0.1% DMSO control. At the highest concentration of selumetinib tested, 92.1 spheroid cell viability was reduced by 81.5% and MM66 by 62.5% when compared with the 0.1% DMSO control. The effect of selumetinib on 2D-cultured 92.1 cells were similar to those observed in 3D; however, for MM66 cells, selumetinib had no effect on reducing cell viability in 2D culture (Fig. 4D). Although it was not possible in this study to examine the penetration of 3D spheroids by selumetinib, the morphology of 92.1 and MM66 spheroids following treatment with this drug suggested that, unlike doxorubicin, which penetrated to the core of the spheroid, selumetinib acted predominantly on the cells close to the surface of the spheroid, leaving an intact viable cell core 48 hours after treatment. The ATP signal at the highest selumetinib concentration, compared to doxorubicin at the highest concentration, is suggestive of this remaining cellular core.

The effects of doxorubicin in 92.1 and MM66 spheroids formed from an initial plating density of 7500 cells/well (Fig. 4) were similar when cells were plated at 5000 and 10,000 cells/well (Supplementary Figure S1). This demonstrates that drug penetration was unaffected by increasing spheroid diameter in 92.1 and MM66 cells up to a maximum of 1144.46 ± 288.62 µm and 1695.28 ± 128.32 µm, respectively, as examined in this study.

## Discussion

In this study, we describe details of PUM cell-derived spheroids and define the methods for generating 3D spheroids from both UM cell lines and PUM for use in downstream drug screening assays. Morphological, immunohistochemical, and genetic analyses of fixed PUM spheroids confirmed that they retain the histological and genetic characteristics of the primary tumor and as such are an important first step in treatment personalization. Development of a drug discovery pipeline has been initiated to measure endpoints such as spheroid cross-sectional area and to use viability endpoint assays to measure ATP synthesis.

Traditionally, modeling of cancer cell biology in an in vitro setting has been confined to 2D cell culture models, which have been used to analyze a range of cell behaviors (e.g., proliferation, migration, invasion) in both drug-treated and untreated cells. However, more recently, researchers have been developing 3D cell culture models that incorporate the physiological TME, allowing them to more closely recapitulate tumor characteristics, with the aim of providing more translatable results.^1^^,^^27^ To establish a 3D UM spheroid model in this study, a range of reported techniques, including hanging drop and poly(2-hydroxyethyl methacrylate) matrix suspension have been tested.^28^ We established that the use of ULA plates seeded with a range of cell densities provided the most robust and reproducible technique to generate uniform-sized spheroids for each UM cell line and PUM cells.^29^ All cell lines ultimately produced uniform-sized spheroids; however, it is important to note that the spheroids differed among each UM cell line and PUM cells, in terms of overall size (measured by cross-sectional area), compactness, and density. Further, the time taken to undergo the spheroid cellular reorganization varied from 4 to 7 days for four UM cell lines (92.1, MP46, OMM2.5, and OMM1), and up to 10 days for two others (MM66 and MP41). This variability in the formation of spheroids by UM cell lines cannot be explained by their underlying genetic profile but is important to consider when designing drug screening studies, such that several parameters, including a minimum cross-sectional area and cell density, are defined as the point at which drug testing should commence. From our initial data, we suggest a minimum cross-sectional area of 1 × 10^6^ µm^2^. Although we were unable to estimate cell density, future studies will use an algorithm present on the Cytation 5 (BioTek UK, Swindon, UK) to determine this. Ultimately, this may require the slower growing cells (e.g., MP46) or cells that compact less rapidly (e.g., MP41, MM66) to be plated at higher densities and/or tested at later time points.

Most of the UM cell lines could be removed from the ULA plates for downstream analyses, with the exception of MP41 and OMM1 spheroids, which disaggregated with pipetting. Such tumor spheroid disaggregation on removal from ULA wells has been reported by other investigators.^30^^,^^31^ It is unclear why this occurs, but it may be caused by differences in extracellular matrix deposition or by the intercellular adhesions between tumor cells. Ivanov and Grabowska^30^ have cleverly designed agarose molds that do not require the tumor spheroids to be removed from the wells for histological embedding, sectioning, or staining; instead, spheroid tissue microarrays are created, allowing for high-throughput analysis of large 3D tumor cell spheroid sample sets. We are currently trialing such molds to examine numerous UM cell line spheroids in parallel in order to undertake multidrug screening*.*

Also novel in the current study is the description of the successful generation of UM spheroids derived from PUM samples, with morphological, immunohistochemical, and genetic characteristics representative of the original patient tumor. In general, the PUM spheroids generated had a low proliferative index, apart from S093, which also showed central necrosis and contained admixed macrophages, which are present in both PUM^32^ and metastatic UM^33^ and are associated with a poor outcome.^34^ Although the largest and least compact of the PUM spheroids was formed by the sample with dense macrophage content, any correlation between this feature and spheroid growth characteristics was not possible to determine in the current study due to the small numbers analyzed. Admixed macrophages and other cells within the PUM are also likely to influence drug uptake and/or drug response as shown in colon cancer spheroids.^35^ Fresh PUM samples are relatively difficult to obtain and should be harvested soon after surgery; when they have been cultured, PUM cells show changes in cellular morphology and molecular characteristics within a few passages.^36^ We recommend the generation of PUM spheroids from early passage cells with associated histological and genetic work-ups, and yet note that ∼30% of PUM may not form 3D spheroids. The reasons for this remain unclear, but we speculate that this could be due to specimens with a very low proliferation rate that in general also fail to grow after the first passage in 2D-culture due to senescence. This clearly has implications for personalized medicine, and further studies are necessary to determine whether UM cells isolated from metastatic lesions have a similar failure rate in 3D culture.

Two other interlinked features of the UM spheroids that we examined in this study were drug penetrance and efficacy, which we investigated through the use of two drugs previously administered in the context of metastatic UM—namely doxorubicin and selumetinib. The two drugs have differing modes of action; doxorubicin acts by intercalating with DNA to cause DNA damage, and selumetinib is an ATP-independent inhibitor of mitogen-activated protein kinase/extracellular signal-regulated kinase 1/2.^37^^,^^38^ For both doxorubicin and selumetinib, entry into the cell is influenced by plasma membrane lipid composition. Doxorubicin and selumetinib have molecular weights of 543.5 and 457.7, respectively, suggesting that the differences in cell viability observed for the two drugs is not merely due to their ability to penetrate the spheroid. Moreover, similar effects of doxorubicin and selumetinib were observed in spheroids of varying size. Instead, we suggest that the mixture of growing and quiescent cells in differing metabolic states that likely exist within the larger spheroids will affect drug efficacy in a manner more akin to that observed *in vivo*.

The aspect of drug penetration was specifically assessed by profiling doxorubicin, which is known to autofluoresce and act as a biocompatible fluorophore with an absorption peak around 488 nm of Ar^+^ laser.^25^ The penetration of doxorubicin into the UM spheroids was easily observed at the higher concentrations, which has previously been seen in other cancer spheroids, such as breast.^27^ Although it was not possible to fully assess the degree of penetrance of selumetinib, cell viability at the periphery of the 92.1 and MM66 spheroids appeared reduced upon imaging; this was confirmed by use of the CellTiter-Glo 3D Cell Viability Assay, which assessed the viability of all cells within the spheroid. This effect of spheroid cell killing occurring at the periphery has recently been reported in squamous cell carcinoma spheroids treated with a microtubule-inhibiting small molecule that was able to slowly work inward to cause the death of cells within the central core.^39^ Methods used to increase drug penetrance into spheroids have also been described, including electrochemotherapy.^40^

With respect to drug efficacy, studies have shown that in 2D cell culture systems potency is enhanced when compared with 3D models,^21^ such that the predicted clinical efficacy in patients is often overestimated.^1^ Interestingly, in our study, doxorubicin had similar effects on 92.1 and MM66 cells grown in 2D or 3D culture irrespective of spheroid parameters such as compactness, growth rate, or cross-sectional area. The results for selumetinib in the MM66 cell line, however, were somewhat unexpected, as selumetinib was more effective in MM66 cells in 3D than in 2D when ATP synthesis was assessed. One possible explanation for this could be more rapid proliferation of the unaffected cells in a 2D setting that is not seen with the more slowly cycling cells in a 3D spheroid. Measuring recovery of cellular growth in 3D spheroid models will, therefore, be of importance when considering residual tumor burden and drug resistance.

Goncalves et al.^8^ also reported the effects of another MEK inhibitor, trametinib, in 3D spheroids of UM cell lines placed in a collagen matrix. They observed an increase in the number of dead cells at the edge of the spheroid following treatment with a single concentration of trametinib, as measured by propidium iodide staining. Shaughnessy et al.^9^ described the formation of multiple spheroids when UM cell lines were placed in a Matrigel matrix, and they used this method to screen the effects of the calcium channel blocker amlodipine. In this latter study, spheroids of differing sizes were produced and then randomly sampled for measurement in the presence or absence of the drug. Cell surface interactions with extracellular matrices are also likely to contribute to drug efficacy and penetration, factors that were not discussed in these papers but which must be considered when developing assay systems for drug discovery and screening.

In conclusion, we have described optimal conditions for the reproducible production of 3D spheroids of uniform size from UM cell lines and PUM samples, as well as their use in drug screening assays. We are of the opinion that the panel of UM cell lines examined in this study can be used to mimic many aspects of the heterogeneous in vivo tumor cell population, such as proliferation, oxygen and nutrient gradients, and cellular interactions when grown in a 3D culture as compared with monolayer cultures. In particular, monitoring of 3D phenotypic parameters, such as spheroid size, shape, and internal features, combined with a variety of endpoint assays, will allow the outcomes of drug action at a multicellular level to be more accurately evaluated. Further development of 3D culture methods may involve incorporation of multiple cell types that mimic the metastatic TME and the use of hydrogels as scaffolds to encapsulate the cells or to serve as mechanical and biochemical cues that more accurately represent the composition of the tumor extracellular matrix and the sequestration of biomolecules within.^41^^,^^42^ Development of these methodologies is ongoing for many tumor types, including UM, and requires a detailed understanding of the TME composition, stiffness, and surface chemical properties. Although these more complex models will ultimately allow a better understanding of UM biology and drug efficacy, their development is time consuming and expensive and poses numerous challenges in terms of microscopy and assay measurements. New imaging modalities such as Cytation 5/7 (BioTek) and Incucyte S3 (Essen Bioscience, Hertfordshire, UK) offer live imaging of spheroids with software packages and complex algorithms for powerful analysis of cellular events in scaffold-free and scaffold-based 3D cell culture models. The use of well-defined 3D models of UM cell lines and ultimately metastatic UM patient material will lead us closer to the goal of personalized medicine.

## Supplementary Material

Supplement 1
