# Supplementary figures and images for: Characterization of Uveal Melanoma Cell Lines and Primary Tumor Samples in 3D Culture
Source: Transl Vis Sci Technol. 2020 Jun 29;9(7):39. doi: 10.1167/tvst.9.7.39 (PMC7414609; doi:10.1167/tvst.9.7.39)

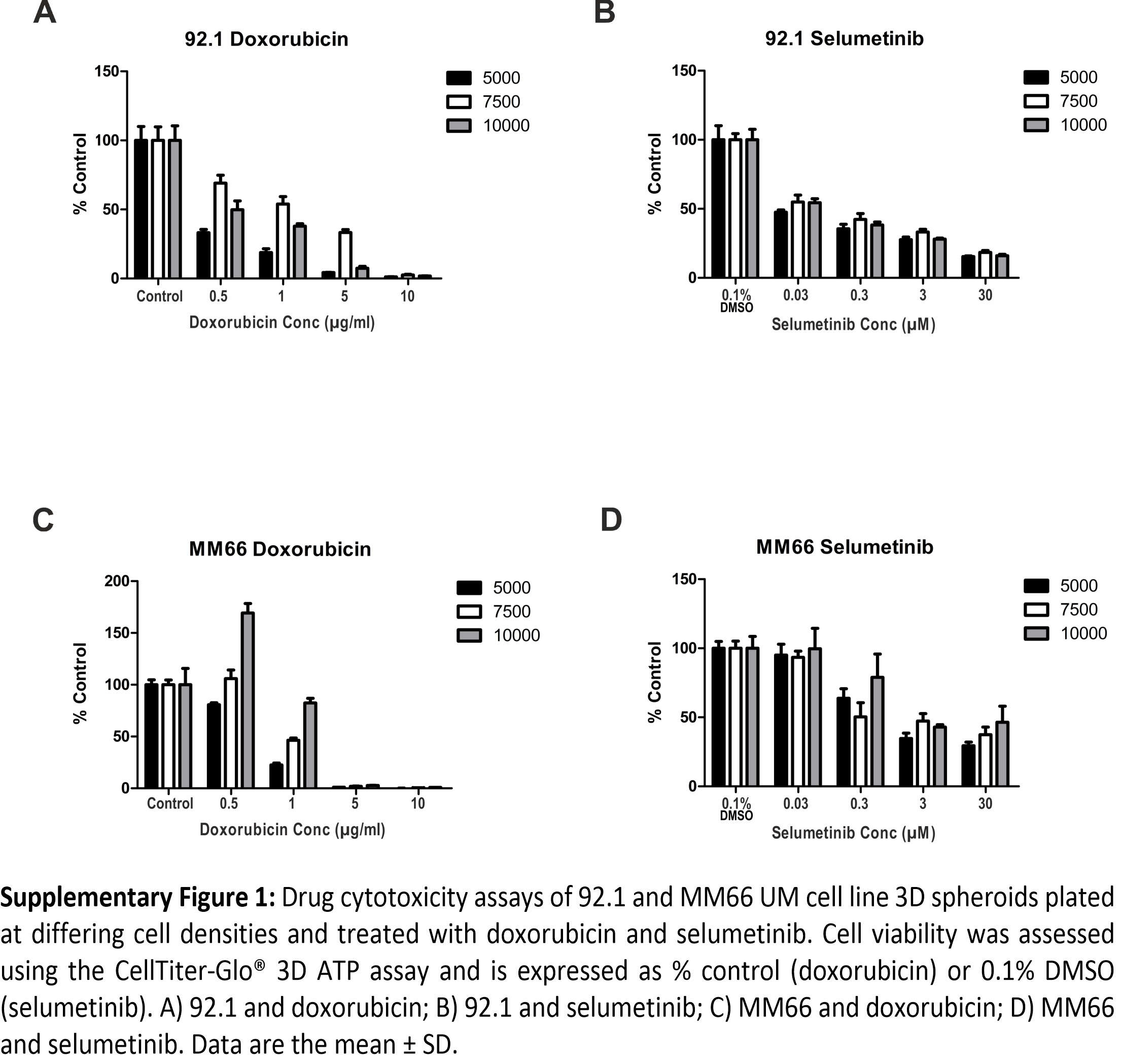

Supplement: Supplement 1 [file tvst-9-7-39_s001.jpg]
